# Treatment Strategies of Gastric Cancer—Molecular Targets for Anti-angiogenic Therapy: a State-of-the-art Review

**DOI:** 10.1007/s12029-021-00629-7

**Published:** 2021-03-24

**Authors:** Magdalena Tyczyńska, Paweł Kędzierawski, Kaja Karakuła, Jacek Januszewski, Krzysztof Kozak, Monika Sitarz, Alicja Forma

**Affiliations:** 1 Department of Human Anatomy, Medical University of Lublin, 20-090 Lublin, Poland; 2 Department of Forensic Medicine, Medical University of Lublin, 20-090 Lublin, Poland; 3 Department of Psychiatry, Psychotherapy and Early Intervention, Medical University of Lublin, Gluska Street 1, 20-439 Lublin, Poland; 4 Department of Conservative Dentistry with Endodontics, Medical University of Lublin, 20-090 Lublin, Poland

**Keywords:** Gastric cancer, Carcinogenesis, Angiogenesis, Anti-angiogenic therapy, Treatment, Molecular target

## Abstract

**Purpose:**

Recent studies have suggested that molecular targets for the anti-angiogenic therapy might constitute a basis for additional therapy in gastric cancer treatment. A vast number of molecules, receptors, pathways, specific interactions, and thus strategies that target gastric cancer angiogenesis specifically have been reported in numerous research articles and clinical trials.

**Methods:**

We conducted a systematic literature review of molecularly targeted treatment strategies in gastric cancer on the following databases—PubMed, Google Scholar, and Scopus—on September 20, 2020. Multiple articles and evaluations were searched for studies reporting newly found and promising molecular anti-angiogenic therapy pathways. Eventually, 39 articles regarding the anti-angiogenic therapy in gastric cancer were included in the final analysis.

**Results:**

As a consequence of the release of the pro-angiogenic molecules from the tumour cells, gastric cancer presents high angiogenic capability. Therefore, potential schemes for future treatment strategies include the decrease of the process ligands as well as the expression of their receptors. Moreover, the increase in the angiogenic inhibitor levels and direct aim for the inner walls of the endothelial cells appear as a promising therapeutic strategy. Beyond that, angiogenesis process inhibition seems to indirectly exaggerate the effects of chemotherapy in the considered patients.

**Conclusions:**

The anti-angiogenic treatment in gastric cancer patients evaluates its significance especially in the early stages of the malignancy. The studies conducted so far show that most of the meaningful angiogenic factors and receptors with the potential molecular pathways should be further evaluated since they could potentially play a substantial role in future therapies.

## Introduction

With more than 990,000 new diagnoses and over 738,000 death cases reported every year, gastric cancer (GC) is estimated to be the fourth most prevalent cancer and currently, it is in the second position in terms of cancer-related deaths [1–3]. The characteristics, as well as the prevalence of GC, significantly differ between sexes and particular world regions. Males are reported to be almost two to three times more susceptible to the onset of GC comparing to females [4, 5]. The epidemiological studies show that more than 50% of the newly diagnosed patients are those from developing countries (Eastern Europe, East Asia, Central, and South America); whereas the lower-risk areas include Southern Asia, East Africa, and North America [6]. Besides, the 5-year survival rate of patients also differs depending on the particular region; in Europe, the survival rate is estimated at 10–30% [7]. Over the last few decades, it was observed that GC incidence rates decreased in most parts of the world. This data, however, concerns only the sporadic intestinal type of GC, whereas the prevalence of the diffuse type of GC has increased [8, 9]. GC is classified according to two major histological classifications—the Lauren classification and the World Health Organization (WHO) classification [10]. GC might develop due to a vast number of risk factors including enhanced exposition to chemical carcinogens and other environmental factors, family history and genetic predispositions, improper diet, excessive alcohol consumption, or Epstein–Barr virus (EBV) infection; however, the infection by *Helicobacter pylori* (*H. pylori*) still remains the major cause of GC induction [11–13]. Several hallmarks of carcinogenesis are crucial in the induction of GC including chronic inflammation, enhanced angiogenesis, or—quite prevalently—the onset of the epithelial–mesenchymal transition (EMT) [14–18].

**Fig. 1:**
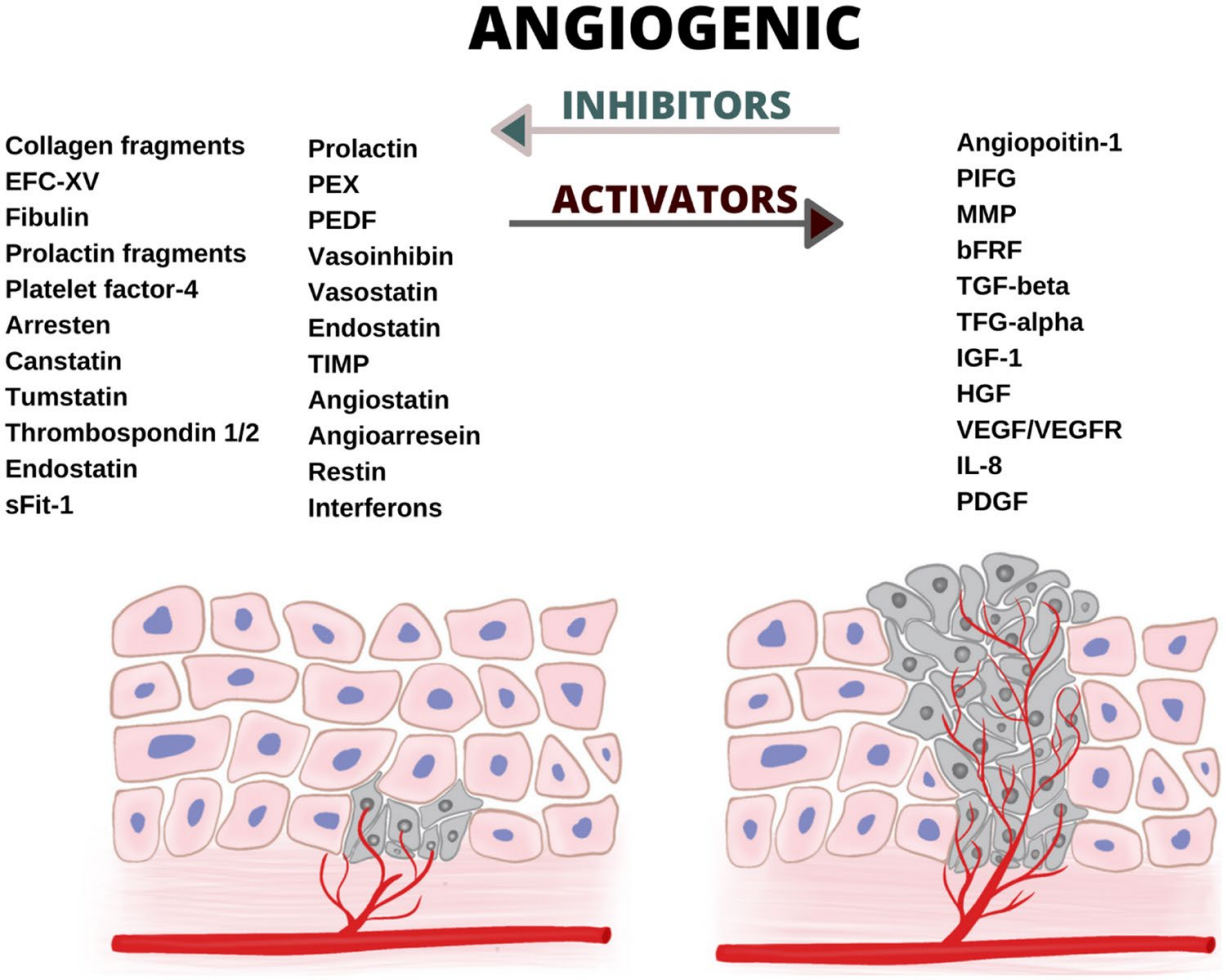
Regulation of angiogenesis and vascular homeostasis through the angiogenic activators and inhibitors. EFC-XV—endostatin-like fragment of type XV, sFit-1—soluble Fms-like tyrosine kinase 1, PEX—C-terminal hemopexin-like domain, PEDF—pigment epithelium-derived factor, TIMP—tissue inhibitors of matrix metalloproteinases, PIGF—placental growth factor, MMP—metalloproteinases, bFRF—basic fibroblast growth factor, TGF-beta—transforming growth factor-beta, TGF-alpha—transforming growth factor-alpha, IGF-1—insulin-like growth factor-1, HGF—hepatocyte growth factor, VEGF/VEGFR—vascular endothelial growth factor/vascular endothelial growth factor receptor, IL-8—interleukin-8, PDGF—platelet-derived growth factor

Carcinogenesis is a multistep process whose progression is highly associated with the angiogenic switch. Angiogenesis is the formation of the new blood vessels from the pre-existing ones and might be either physiological or pathological depending on the conditions (Fig. 1).

Angiogenesis enables cancer cell metastasis, as well as the expansion of the cancerous tissues since the newly formed vessels provide nutrition, proper oxygenation, and supply of the crucial growth factors [19–21]. Pathological angiogenesis is highly uncontrolled due to the dominance of the pro-angiogenic factors [22, 23]. Tumour blood vessels are often inefficient because of the disturbed vascular structure—they tend to form chaotic networks with irregular branching. Furthermore, the blood perfusion through the tumour blood vessels is not continual and bidirectional blood flow might occur [24, 25]. Tumour cells release a significant number of molecules that induce the growth of new vasculature, promoting angiogenesis. Studies have shown that GC cells present a high angiogenic potential; the major pro-angiogenic factors include the vascular endothelial growth factor (VEGF) family with placental growth factor (PIGF) [26, 27], the angiopoietin/Tie-cascade [28], fibroblast growth factors (FGF) [29], hypoxia-inducible factor 1 and 2 (HIF-1 and HIF-2) [30], tryptase [31], and integrins [32, 33].

Currently, there are multiple strategies for GC treatment including a vast number of surgical techniques, chemotherapy, immunotherapy, and targeted therapies including those focused on angiogenesis inhibition for example. The potential strategies of GC treatment include the decrease of the proangiogenic ligands and the expression of their receptors and the increase of the angiogenic inhibitor levels, as well as targeting the inner walls of the endothelial cells (EC) directly [34]. Additionally, the inhibition of the angiogenesis seems to intensify the effects of chemotherapy indirectly, probably because of the vessels’ normalization and more effective delivery of the chemotherapeutic agents [35, 36]. The purpose of this review is to evaluate the significance of the anti-angiogenetic treatment in GCs patients, especially in early (perioperative and neoadjuvant) stages of cancer. We summarized the most significant angiogenetic factors and receptors together with potential molecular pathways, their effects, and possible involvement in the anti-angiogenic therapy of GC.

## Biomarkers of Angiogenesis

Since angiogenesis constitutes one of the most crucial hallmarks of carcinogenesis, numerous new therapeutic agents that target this process specifically are currently under investigation. Biomarkers of angiogenesis and their expression provide knowledge about the efficacy of therapy as well as an insight into such aspects as appropriate dose or duration of anti-angiogenic inhibitors. A vast number of those biomarkers are currently used for clinical purposes; however, the aim of further research is to provide an insight into which specific one or which combination might be the most effective in the control of the anti-angiogenic therapy. In this paragraph, we have briefly summarized several most important biomarkers in terms of angiogenesis and further control of the anti-angiogenic therapy.

### Vascular Endothelial Growth Factor Family

VEGF is one of the most significant factors involved in the stimulation of angiogenesis during gastric carcinogenesis [37]. The VEGF family consists of 7 major subtypes including VEGF-A, VEGF-B, VEGF-C, VEGF-D, VEGF-E, VEGF-F, and PIGF [38, 39]. VEGF-A released by the gastric cancer cells is considered to be the major pro-angiogenic factor involved in gastric tumorigenesis [40]. Moreover, the expression of VEGF-A increases the number of vessels in both - the intestinal and the diffuse type of GC [41]. Studies demonstrated a worse overall prognosis in patients with VEGF-positive tumours compared to the VEGF-negative ones [42].

The binding of VEGF-A to its receptor—VEGFR-2—is considered to be the most crucial activator of the angiogenic pathways [43]. The binding promotes a cascade of signals that result in the proliferation and migration of the ECs, enhanced vascular permeability, modification of gene expression, and activation of the Ras pathway [44, 45]. Contrarily, the role of VEGFR-1 is more intricate and not fully deciphered yet. A soluble form of VEGFR-1 can act as a bait receptor and prevent VEGF-A/VEGFR-2 binding, which inhibits the activation of the signalling pathway. However, there is evidence that VEGFR-1 plays an essential role in the progression of angiogenesis [46]. VEGFR-3 stimulates lymphangiogenesis and does not bind to the VEGF-A [47].

The neuropilins 1 and 2 (NRP1 and NRP2) are involved in the cell guidance and the enhanced binding of VEGF to its signalling receptors [48]. A vast number of other factors present similarity to VEGF-A in terms of the stimulation of the angiogenesis and those include PIGF, FGF, VEGF-C, VEGF-D, angiopoietin, HIF-1α and HIF-2α, integrins, and PDGF [49, 50].

Overexpression of another member of the VEGF family—PIGF—leads to pathological angiogenesis [51]. Furthermore, it was shown that PIGF overexpression correlates with metastases to lymph nodes, tumour growth, and poor overall survival in GC [52].

### Resistin-like Molecule-α

Resistin-like-molecule-α (RELM-α) is a protein that plays a significant role in GC progression despite being a marker for anti-inflammatory macrophages [53]. It was reported that RELM-α expression is associated with tumour size and staging [54]. Besides, RELM-α activates the members of the VEGF family by activating the NF-κB-MMP-9/VEGF pathway, enhancing the process of angiogenesis [55]. RELM-α is now considered a new and promising biomarker in GC [5].

### Angiopoietins

During physiological conditions, angiopoietins (Angs) are involved in the embryonic and postnatal angiogenic processes. So far, the most examined members of this family include angiopoietin-1 (Ang-1) and angiopoietin-2 (Ang-2), which are highly overexpressed in gastric cancerous tissues [56]. Ang-1 and Ang-2 are the ligands for the tyrosine-protein kinase receptor—Tie-2. Ang-1/Tie-2 binding stabilizes the vessels due to the recruitment of the pericytes [57]. On the contrary, Ang-2 antagonizes Tie-2 preventing the maturation of Ang-1, which leads to the suppression of vasculature growth [58]. Furthermore, the disparities between Ang-1 and Ang-2 levels might be associated with the severity of the pro-angiogenic process.

### Platelet-derived Growth Factor-β Family

PDGF-β is a crucial component of the tumour progression, as it induces both angiogenesis and EMT, which are highly relevant regarding the alterations within the GC microenvironment [59]. It was shown that PDGF-β and VEGF are secreted simultaneously in gastric cancerous tissues, though PDGF-β is believed to be more crucial in the maintenance of the blood vessels in the intestinal type of GC [60]. In addition, PDGF-β is essential in the migration, proliferation, and adhesion of the endothelial progenitor cells, which are required for both neovascularization and re-endothelialization [61, 62].

### Interleukin-8

IL-8 was originally described as a chemokine whose main role was to attract the polymorphonuclear inflammatory leukocyte infiltrate acting on CXCR1/2 [63]. Recently, it was found that various tumours often overproduce this chemokine, which presents the pro-tumoural characteristics in various malignancies [64]. IL-8 is involved in the angiogenesis, survival signalling for cancer stem cells, and attraction of the myeloid cells, as well as delivery of the local growth factors [65]. Moreover, IL-8 is highly produced by the tumour cells; thus, its serum concentration is significantly associated with tumour growth [66]. Therefore, IL-8 serum concentration is considered to be a useful pharmacodynamic biomarker in the early detection of the immunotherapy response in GC patients [67].

### MicroRNA

MicroRNAs (miRNAs) constitute a group of small, single-stranded, non-coding RNAs that regulate the expression of various genes at the point of post-transcription [68]. Hence, miRNAs are involved in various cellular functions such as proliferation, apoptosis, regulation of embryonic stem cell advancement, and cancer cell invasion [69]. The latest studies have shown that miRNAs are remarkably stable in the blood flow, besides their concentration might present the efficacy of the therapy; this discovery shows that they might be reliable biomarkers of GC therapy [70]. Several studies showed that miRNAs can regulate tumour angiogenesis through targeting both the pro- and antiangiogenic factors, such as RTK signalling protein, HIF, VEGF, and epidermal growth factor (EGF) [71]. miRNA-126 has an essential role in regulating the angiogenesis processes; elevated expression of miRNA-126 is associated with the increased VEGF-A signalling in ECs. Therefore, miRNA is believed to be a promising biomarker of the anti-angiogenic therapy of GC [72, 73].

### Circulating Tumour Cells and Free Nucleic Acid

Circulating tumour cells (CTCs) and free nucleic acid (CTNA) are metastatic cells that are released into the bloodstream by the primary tumour cells; thus, they are involved in the recognition of the hematogenous metastasis [74]. CTCs and CTNA detection in the peripheral blood is a potential strategy for the non-invasive diagnosis and estimation of GC patients’ response to treatment therapies [75, 76]. The conducted studies suggest that patients with a low baseline CTC count or drop of the CTC amounts after the first cycle of chemotherapy might substantially benefit from the palliative chemotherapy [77]. Therefore, CTC count might be a good chemotherapy-supervising marker and a prognostic marker for patients receiving palliative chemotherapy [49].

## Currently Available Treatment Strategies of Gastric Cancer

The treatment of GC depends on the type, severity, and stage of GC—treatment modalities differ in early (EGC) and advanced (AGC) stages of cancer. Nowadays, the most common radical treatment strategy of GC is surgery—gastrectomy [78]. However, there are novel methods that continually become more advanced and more frequently used, such as neoadjuvant chemotherapy, radiotherapy, immunotherapy, and molecularly targeted therapies [78–80].

### Surgical Treatment

The surgical treatment of GC constitutes the major and most frequently used treatment strategy in GC patients; two major types—open and minimally invasive laparoscopic gastrectomies—are distinguished [81, 82]. Laparoscopic gastrectomy can be performed by either the operator or a robot—so-called robotic laparoscopy [81]. The Japanese Gastric Cancer Association distinguishes several types of gastrectomies including total gastrectomy, distal gastrectomy, pylorus-preserving gastrectomy, proximal gastrectomy, segmental gastrectomy, local resection, and non-resectional surgery such as bypass surgery or gastrostomy [82]. Best et al. [83] showed no difference in the short-term mortality rates between laparoscopic and open gastrectomies. The laparoscopic treatment includes the totally laparoscopic distal gastrectomy (TLDG) and the laparoscopic-assisted distal gastrectomy (LADG). Hyui et al. [84] showed that there is no remarkable difference in surgical outcomes and postoperative complications between TLDG and LADG. It was reported that TLDG was preferably chosen due to the lower percentage of postoperative pain and quicker recovery. Surgery treatment also includes the dissection of the lymph nodes [85]. Regional lymph nodes of the stomach are classified into the stations from 1 to 20 plus stations 110, 111, and 112 [86]. The nodal resection as a part of GC treatment can be divided by the D-level criteria depending on the type of gastrectomy [82, 86]. The extent of lymphadenectomies performed worldwide differs significantly between the countries [87].

### Chemotherapy, Adjuvant Chemoradiotherapy, and Neoadjuvant Chemotherapy

Chemotherapy, as a perioperative treatment, has been improved significantly over the recent years [88]. The survival rate of patients treated with chemotherapy increased compared to patients treated applying the surgical resection alone [89, 90]. In the case of GC, chemotherapy is preferred in patients with AGC unlike in the case of patients with EGC patients, who are preferred to be treated applying surgical methods [91]. Usually, chemotherapy does not provide a complete cure, however, in the case of patients with unresectable GC, the obtained median survival time was estimated at 6–13 months; adjuvant chemotherapy also seems to be reasonable [82]. Macdonald et al. [92] compared surgical treatment alone versus surgical treatment with fluorouracil and leucovorin treatment combined with radiotherapy. Eventually, the overall survival rate while applying only surgery alone was 26 months, and in investigated adjuvant chemoradiotherapy, it was prolonged up to 36 months [92]. Also, Guimbaud et al. [93] in phase III of their prospective, multicenter, randomized trial compared epirubicin, cisplatin, and capecitabine (ECX) to fluorouracil, leucovorin, and irinotecan (FOLFIRI) as the first-line treatment of AGC and gastroesophageal junction (GEJ). After a median of 31 months, follow-up time-to-treatment failure (TTF) was significantly longer for FOLFIRI in comparison to TTF of ECX (5.1 versus 4.2 months) [93]. The authors announced that FOLFIRI as first-line treatment should be considered as a backbone regiment for targeted treatment agents and, in this case, should be explored [93]. Surgical treatment and dissection of the tumour can cause the activation of the tumour cell growth–stimulating factor and lead to immediate growth of GC tumour. Moreover, it can entail the production of anti-chemotherapy agents [94]. Thus, the target of neoadjuvant chemotherapy (NAC) is to down-stage tumour and to eliminate potential metastases which can let R0 resection [79]. NAC as a worldwide-accepted treatment was approved by MAGIC randomized trial 903. Terashima et al. [95] in their phase III randomized trial take on targeting the efficiency of NAC considering morbidity, morality, and surgical aspect in stage 3 and stage 4 GC. In their results, there was no major growth of morbidity and mortality registered; however, the time of surgical procedure was significantly shorter (median time equal to 240 and 255 min for those with and without NAC, respectively) [95].

### Radiotherapy

Radiotherapy is a treatment modality that is frequently combined with chemotherapy in the so-called chemoradiotherapy. After the American SWOG/INT0116 clinical trial, radiotherapy currently constitutes a standard treatment strategy just after the radical gastrectomy in many Oncological Units [96, 97]. However, radiotherapy is also developed as a palliative, first in the 1960s, and adjuvant to neoadjuvant treatment of GC [78, 98]. Patients with not fully resected tumours are treated palliatively. The recurrent or locally advanced tumour treated by radiotherapy does not give a well-tolerated modality to palliate bleeding, obstruction, or pain, but it is also effective [98]. There are few methods of radiotherapy such as proton beam radiotherapy (PBT) or photon radiotherapy (RT) [98]. There are also some techniques of treatment simulation such as 2-dimensional simulation or 3-dimensional simulation [99, 100]. During the radiation therapy treatment, patients are exposed to 45 to 50.4 Grey (Gy) in total, which can eventually result in serious adverse reactions [78, 101]. Gao et al. [102] showed that intraoperative radiotherapy (OIRT) did not prolong the overall survival of GC patients but instead, and it had favourable effects in stage II and stage III tumours.

### Immunotherapy

Immunotherapy with checkpoint inhibitors is a novel method that has entered the clinical practice as the human immune system can be stimulated to identify the malignant tumours by the immune surveillance and ultimately inhibit the tumour growth [103, 104]. Nowadays, immunotherapy is targeted at the expression of the PD-1 checkpoint receptor ligand, PD-L1, and the tumours with microsatellite instability (MSI-H) [103, 105]. PD-1 is expressed on the activated T-lymphocytes (primarily to the intra-tumoural antigen–specific CD8+ T lymphocytes), and binds its ligands—PD-L1 and PD-L2—on the tumour cells which leads to exhaustion, anergy, or apoptosis of that lymphocyte and let the growth and development of the tumour [106–110]. Thus, blocking those immune checkpoints stimulates the T lymphocytes against GC cancer cells by avoiding their binding to the ligands [106]. In the USA, the first and only inhibitors approved by the Food and Drug Administration (FDA) in MSI-H tumours are pembrolizumab and nivolumab (primarily used in Japan). Several others are currently investigated in the clinical trials—ramucirumab (phase I), leucovorin (phase II), avelumab (phase III); the last one was accepted by the European Medicines Agency (EMA) [111]. According to Kamath et al. [106], pembrolizumab provided a promising response at about 12–22% in third-line treatment of locally advanced and metastatic GC.

### Molecularly Targeted Therapy

Molecularly targeted therapy is a treatment modality that is based on the growth, cell cycle, apoptosis, angiogenesis, and invasion [78, 112, 113]. Main treatment strategies include the epidermal growth factor receptor (EGFR), VEGF, matrix metalloproteinase (MMP), erythroblastic leukemia viral oncogene homolog 2 (HER2—a member of the EGFR family), and RTK/RAS pathway [78, 80, 114].

EGFR is the receptor whose activation stimulates the intracellular pathways that lead to enhanced proliferation, migration, and angiogenesis. Thus, blocking this receptor inhibits tumour proliferation [78]. There are several agents that block EGFR such as anti-EGFR monoclonal antibodies or EGFR tyrosine kinase inhibitors (EGFR-TKI) [78]. Cetuximab is considered to be a good medicament in the untreated GC [115]. Pinto et al. [115] in their phase II trial compared cetuximab plus docetaxel and cisplatin to docetaxel and cisplatin alone. The rate of response was 41%, and it was higher than in docetaxel and cisplatin alone treatment [115, 116].

VEGF belongs to the family of cytokines that regulate angiogenesis [117]. This family consists of 7 main subtypes including VEGF-A, VEGF-B, VEGF-C, VEGF-D, VEGF-E, VEGF-F, and PlGF [118]. There were two clinical phase III trials—REGARD and RAINBOW—both focused on ramucirumab (VEGFR-2 antibody) on previously treated AGC [119, 120]. However, despite the promising results, those agents do not inhibit the progression of GC; however, they can prolong survival time, unfortunately, only in months [117].

The MMP family consists of few members, inter alia MMP-1, -2, -7, -9, and -12 [78]. MMPs degrade cellular compounds, which in consequence leads to growth, progression, and metastases of the tumour [121]. Marimastat is the MMP inhibitor of the abovementioned MMPs; combined with 5-fluorouracil (5-FU), this drug is considered to be a valuable anticancer treatment strategy [122].

An HER-2 signalling pathway is another way of treatment that is continually investigated and improved [114]. There are few ongoing trials (phase II and III) of trastuzumab: NCT02205047 (INNOVATION)—phase II; NCT01774786 (JACOB)—phase II; and RTOG 1010—phase III. Trastuzumab-based therapies are continually improved especially in the case of HER2-positive GCs [114, 123].

## Anti-angiogenic Therapy—Potential Molecular Targets and Drugs

Angiogenesis constitutes one of the major processes that enable metastasis of GC cells leading to the growth of the secondary tumours. Therefore, the anti-angiogenic drugs are considered to be potential drugs that might prevent the formation of pathological vessels eventually repressing tumour growth and metastases. Most of the anti-angiogenic drugs primarily target the VEGF pathway since it plays a crucial role in the induction and progression of angiogenesis. It was demonstrated that anti-VEGF tyrosine kinase inhibitors (sunitinib, sorafenib, apatinib, trebananib, and regorafenib) combined with chemotherapy significantly improve the overall survival rates as well as the progression-free survival of patients with advanced GC [124–126]. Generally, the plasma levels of the angiogenic factors such as VEGF or Angs are believed to be associated with the survival rates of GC patients [127]. One of the IgG1 monoclonal antibodies against VEGFR-2, ramucirumab, is currently recommended as the second-line treatment of GC according to the RAINBOW and REGARD trials; besides, a combination of ramucirumab with paclitaxel is also very effective [128–130]. Ramucirumab has been approved by the FDA as the first drug used in patients with advanced GC after the unsuccessful chemotherapy. Except for VEGFs and Angs, other factors such as insulin-like growth factor-1 (IGF-1) are involved in angiogenesis [131]. It was demonstrated that combined treatment that targets VEGF and IGF-R1 at the same time might be promising in GC treatment. Besides, VEGF production might be suppressed while administrating (-)-epigallocatechin-3-gallate (EGCG) either alone or combined with docetaxel [132, 133]. In the HGF-VEGF axis, angiogenesis could thus also be suppressed by the expression of miR-26a/b [134].

Luteolin is one of the Chinese herbs belonging to the flavonoids which was described to play a role in the inhibition of the tumour cells proliferation and migration as well as suppression of the epithelial-mesenchymal transition (EMT) in the tumour microenvironment [135]. Except that, it was suggested that luteolin might act as a potential suppressor of angiogenesis and the formation of the vasculogenic mimicry tubes by inhibiting the Notch1-VEGF signalling pathway [136]. Another member of the flavonoids, deguelin, inhibits apoptosis and angiogenesis (by targeting VEGF and HIF-1α) in the GC microenvironment [137]. Zerumbone, derived from *Zingiber zerumbet*, was also reported to inhibit angiogenesis by targeting the NF-κB pathway and eventually downregulating VEGF expression [138]. A member of the plant stress hormones belonging to the jasmonate family—methyl jasmonate exerts anti-tumour properties; it also suppresses VEGF expression in a time-dependent manner primarily due to the downregulation of the metalloproteinase-14 (MMP-14) [139]. Xiaotan Sanjie decoction is widely used in traditional Chinese Medicine, and Shi et al. showed that it might significantly downregulate VEGF-A, VEGFR-1, with VEGFR-2, along with VEGF-A mRNA levels primarily by regulating IL-8 levels [140].

Currently, the first-line treatment of GC includes the administration of 5-fluorouracil (5-FU), and it was suggested that its precursor—capecitabine—similarly to 5-FU might reduce VEGF expression in the GC xenografts [141]. It was observed that the metastatic potential of GC could be potentially inhibited by the usage of interleukin-1 receptor antagonist (IL-1RA), which at the same time inhibits the IL-1α/VEGF signalling pathway lowering VEGF expression and further tube formation [142]. Targeting another pathway associated with angiogenesis by the usage of AT1R antagonist—TCV-116—similarly represses tumour growth by inhibiting angiogenesis [143]. Similar results were obtained while applying recombinant human endostatin; however, in this case, except for inhibiting angiogenesis, also the apoptosis of GC cells was observed [144]. Aminoguanidine, an iNOS-selective inhibitor, and NK4, an HGF antagonist, were also shown to play a role in inhibiting angiogenesis in GC patients [145, 146]. A downregulation of the p-ERK–c-Fos–HIF-1α–VEGF pathway by AZD6244 (selective MAPK-ERK kinase inhibitor) represses further angiogenesis progression according to Gao et al. [147]. The HIF-1α–SIRT1 pathway is also suppressed by forkhead transcription factors of the O class 1 (FOXO1), which inhibits the growth of a gastric tumour and prevents vascularization and overexpression of other angiogenesis-related molecules [148, 149]. Another angiogenesis inhibitor—NM3—combined with oxymatrine showed to be effective in suppressing the growth of GC cells in *in vivo* studies [150]. There is an increasing interest in RNA interference (RNAi)–mediated inhibition of such factors as leucine-rich repeat-containing G protein-coupled receptor 5 (Lgr5), or heparanase which seems to be a promising therapeutic method to abolish gastric angiogenesis [151, 152].

## Conclusions

Anti-angiogenic therapy currently constitutes only an additional therapeutic option for patients with the angiogenic phenotype of GC, which might prolong the survival rate of patients as well as increase their quality of life. Even though the anti-angiogenic therapy seems to be favourable and successfully used for clinical purposes, further prospective randomized studies are needed for the evaluation of the potential drugs used as a part of this therapy. One of the therapeutic challenges is a situation when the advanced aggressiveness of GC induces further resistance to anti-angiogenic treatment. Moreover, there is always a risk that multitargeted therapy might constitute a too expensive approach depending on the possibilities of particular medicinal facilities. However, significantly better outcomes are observed in the case of patients that are treated with targeted molecular therapy and anti-angiogenic treatment. Therefore, an increasing number of newly discovered pathways and molecules that interact with one another might enhance the development of new therapeutic agents that can suppress the progression of GC angiogenesis. There is still further need to examine the potential molecular targets described in this review, especially in clinical trials and prospective randomized studies, especially taking into consideration the patients with different histological and molecular types of GC.

## Abbreviations

AGC: advanced stage of gastric cancer
Angs: angiopoietins
Ang-1: angiopoietin-1
Ang-2: angiopoietin-2
bFRF: basic fibroblast growth factor
CTCs: circulating tumour cells
CTNA: free nucleic acid
EBV: Epstein-Barr virus
EC: endothelial cells
ECX: epirubicin, cisplatin, and capecitabine
EFC-XV: endostatin-like fragment of type XV
EGC: early stage of gastric cancer
EGCG: epigallocatechin-3-gallate
EGFR: epidermal growth factor receptor
EGFR-TKI: EGFR tyrosine kinase inhibitors
EMA: European Medicines Agency
EMT: epithelial-mesenchymal transition
FDA: Food and Drug Administration
FGF: fibroblast growth factors
FOLFIRI: fluorouracil, leucovorin, and irinotecan
FOXO1: forkhead transcription factors of the O class 1
GC: gastric cancer
GEJ: gastroesophageal junction
Gy: Grey
HER2: erythroblastic leukemia viral oncogene homolog 2
HGF: hepatocyte growth factor
HIF-1: hypoxia-inducible factor 1
HIF-2: hypoxia-inducible factor 2
*H. pylori*: Helicobacter pylori
IGF-1: insulin-like growth factor-1
IL-1RA: interleukin-1 receptor antagonist
IL-8: interleukin-8
LADG: laparoscopic-assisted distal gastrectomy
LGR5: leucine-rich repeat-containing G protein-coupled receptor 5
miRNAs: microRNAs
MMP: metalloproteinases
MSI-H: microsatellite instability
NAC: neoadjuvant chemotherapy
NRP1: neuropilin 1
NRP2: neuropilin 2
OIRT: intraoperative radiotherapy
PBT: proton beam radiotherapy
PD-L1: PD-1 checkpoint receptor ligand
PD-L2: PD-2 checkpoint receptor ligand
PDGF: platelet-derived growth factor-β
PEDF: pigment epithelium-derived factor
PEX: C-terminal hemopexin-like domain
PIGF: placental growth factor
RELM-α: resistin-like-molecule-α
RNAi: RNA interference
RT: radiotherapy
sFit-1: soluble Fms-like tyrosine kinase 1
TGF-alfa: transforming growth factor-alpha
TGF-beta: transforming growth factor-beta
TIMP: tissue inhibitors of matrix metalloproteinases
TLDG: totally laparoscopic distal gastrectomy
TTF: time-to-treatment failure
VEGF/VEGFR: vascular endothelial growth factor/vascular endothelial growth factor receptor
WHO: World Health Organization
5-FU: 5-fluorouracil
